# Drag reduction through self-texturing compliant bionic materials

**DOI:** 10.1038/srep40038

**Published:** 2017-01-05

**Authors:** Eryong Liu, Longyang Li, Gang Wang, Zhixiang Zeng, Wenjie Zhao, Qunji Xue

**Affiliations:** 1 Key Laboratory of Marine Materials and Related Technologies, Zhejiang Key Laboratory of Marine Materials and Protective Technologies, Ningbo Institute of Materials Technology and Engineering, Chinese Academy of Sciences, Ningbo, 315201, China

## Abstract

Compliant fish skin is effectively in reducing drag, thus the design and application of compliant bionic materials may be a good choice for drag reduction. Here we consider the drag reduction of compliant bionic materials. First, ZnO and PDMS mesh modified with n-octadecane were prepared, the drag reduction of self-texturing compliant n-octadecane were studied. The results show that the mesh modified by ZnO and PDMS possess excellent lipophilic and hydrophobic, thus n-octadecane at solid, semisolid and liquid state all have good adhesion with modified mesh. The states of n-octadecane changed with temperature, thus, the surface contact angle and adhesive force all varies obviously at different state. The contact angle decreases with temperature, the adhesive force shows a lower value at semisolid state. Furthermore, the drag testing results show that the compliant n-octadecane film is more effectively in drag reduction than superhydrophobic ZnO/PDMS film, indicating that the drag reduction mechanism of n-octadecane is significantly different with superhydrophobic film. Further research shows that the water flow leads to self-texturing of semisolid state n-octadecane, which is similar with compliant fish skin. Therefore, the compliant bionic materials of semisolid state n-octadecane with regular bulge plays a major role in the drag reduction.

Learning from nature give us important information on develop new materials with superior performance. For example, nature has created various ways in drag reducing, such as the efficient movement of dolphins, sharks or other fish. The highest swimming speed of dolphin is not more than 20 km/h according to the biomechanical calculation, but the actual speed is up to 40 km/h^1^. During swimming, the surfaces of fish is directly contact with water, thus, the compliant skins or mucus on the fish surfaces plays a major role in drag reduction. Thus, learning from nature, learning from such as dolphin, shark or fish becomes the main direction of the drag reduction research^2^,^3^,^4^.

It is well known that drag is the force required to move an object through a fluid or a fluid through a device, which occurs due to the physical dimensions of the object obstructing and altering the flow of the fluid^5^,^6^,^7^,^8^. Therefore, surface of fish, such as shark skin, dolphin skin or mucus is a model from nature for drag reduction. Microstructure shows that the shark skin is covered by very small individual dermal dentils ribbed with longitudinal grooves. These grooves make the water moving efficiently over the surface, resulting in the drag present on a smooth surface reduction^9^,^10^. In addition, the sector-like scales of fish with diameters of 4~5 mm are almost covered by papillae with a size of 100~300 μm in length and 30~40 μm in width, which is also an inspired design, adaptation, or derivation from nature^11^,^12^. Thus, design and fabrication of bionic materials based on nature is an effective way to reduce drag.

Firstly, the streamline shape of dolphin or shark is considered as the main reasons for drag reduction, but the drag testing results of dolphin shape liked model indicates that the drag is nine times than dolphin^13^,^14^. Further research shows that the compliant dolphin skin can respond to the water flow during swimming, and then reducing the drag^15^,^16^,^17^. Kramer’s tried to mimic the dolphin’s epidermis and claimed drag reduction of as much as 60%. Carpenter reports the results of optimizing a rather simple plate-spring coating, it leads to a drag reduction of 36% at the typical dolphin’s sustained speed and 20% at burst speed^8^,^18^,^19^,^20^. Those results all show that the drag reduction of dolphin like coating is about 30–50%. According to above results, Kramer uses rubber to make a compliant ship model, the maximum drag reduction coefficient is up to 50%^21^. The research of Blick shows that the relative motion of compliant surface and water flow change the fluid boundary layer. The thickness of the fluid boundary layer increases with compliant surface, the transition of laminar into turbulence flow delays, the velocity gradient and shear force decreases, thus, these change of boundary layer reduce the drag^18^,^22^,^23^,^24^. Sam indicates that regular bulge forms on the dolphin skin during the dolphin swimming, and many micro-vortex appeared on the dolphin skin with regular bulge. The sliding friction of water flow can be replaced by rolling friction, thus reducing the drag of dolphin during swimimg^2^,^25^,^26^. Thus, learning from dolphin, shark or fish, the design and application of compliant bionic materials may be a good choice for drag reduction.

In this study, stainless steel mesh modified by ZnO and polydimethylsiloxane (PDMS) was used as the matrix and n-octadecane was selected to simulate the compliant dolphin skin, due to that the melting process of n-octadecane pass three states: solid, semisolid and liquid state^27^,^28^. In semisolid state, n-octadecane was composed of melting liquid area and unmelted solid area, which was similar with the dolphin skin containing regular bulge^29^,^30^,^31^. Thus, the drag reduction of self-texturing ZnO/PDMS/n-octadecane film at different states was investigated, and the drag-reduction mechanism of compliant bionic materials was researched.

## Experimental Approach

### Materials

Stainless steel meshes with pore sizes of 40 μm * 40 μm were selected as the matrix material. Zinc chloride (AR), Ammonium chloride (AR), Ammonia (AR), Urea (AR), Ethyl acetate (AR), and n-octadecane were purchased from Aladdin Reagent and used as received without further purification. SYLGARD® 184 silicone elastomer was purchased from Dow Corning Corporation.

### Preparation of ZnO film

A piece of steel mesh (18 × 9 cm) was ultrasonically cleaned in ethanol and deionized water for 30 min to remove the oils stained on surface. After cleaning, the mesh was dried in oven and then immersed in a solution to prepare ZnO film. The process was followed as listed: 2.9749 g zinc chloride was dissolved in deionized water firstly, 0.10689 g ammonium chloride, 0.6004 g urea, 5 ml ammonia, and 95 ml deionized water was added into the solution one by one, and then the mesh was added at 90 °C for 1 h. Finally, the modified mesh was cleaned in deionized water and dried at 90 °C to form ZnO film on mesh matrix.

### Preparation of ZnO/PDMS film

SYLGARD® 184 silicone elastomer (Dow Corning Corporation), a transparent PDMS (polydimethylsiloxane) was used to modify the ZnO film on stainless steel mesh. The elastomer was first prepared by 1 g resin and 0.1 g curing agent (in weight), and then 100 ml ethyl acetate was added. The ZnO modified mesh was immersed in PDMS solution and then dried in oven, the process repeats ten times to form ZnO/PDMS film on mesh matrix.

### Preparation of ZnO/PDMS/n-octadecane film

N-octadecane was used to fill the ZnO film and ZnO/PDMS film. After modified by ZnO and PDMS, the liquid N-octadecane was dropped into the mesh to form ZnO/PDMS/n-octadecane film on the mesh matrix. The change of n-octadecane during melting process was used to simulate the compliant dolphin skin.

### Characterization

The microstructure of ZnO film on mesh were characterized by SEM (FEI Quanta 250 FEG, U.S.). The surface contact angle, advancing angle, receding angle and hysteresis of contact angle was detected by a contact angle meter (OCA20, Germany). During testing, the volume of water droplets was 3.0 μL, and it was dropped onto the surfaces carefully. After testing, five measurements were performed and averaged.

The adhesive behaviors of water and n-octadecane on the above three films were measured by dynamic CA-measuring device, the testing process was shown in Fig. 3. Firstly, water or n-octadecane droplet with a size of 3.0 μL was placed on a metal circle with a diameter of 2 mm, which was connected to microbalance detector by a metal thread. Then the samples was placed on a movable stage, derived the samples upwards or downwards at a set speed. During testing, the samples were moved to contact with the suspended droplet first and then further moving up to ensured sufficient contact for water adhesion to samples. During testing, the change of weight and distances of the samples and water droplet in the contact-detachment process was plotted on the microbalance versus the position of the stage.

**Figure 1:**
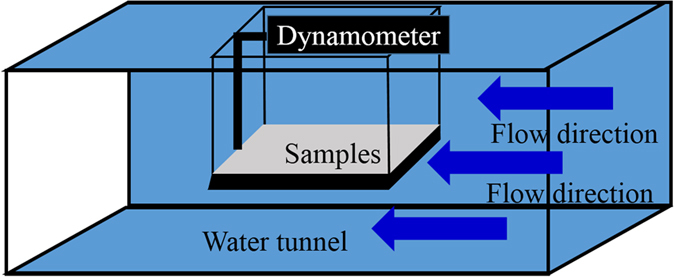
Schematic of the drag testing equipment.

Drag reduction of different films was tested by water tunnel, and the schematic of the drag testing equipment was shown in Fig. 1. It can be seen that the observation area was focused on a section of the water tunnel with length 80 cm, width 10 cm, and height 10 cm. Before testing, a plate samples with a size of 18 cm * 9 cm * 0.4 cm are placed on the sample stage firstly, and then the water flow was induced into water tunnel by an electric pump. During testing, a dynamometer was used to measure the drag of the sample, a speed adjustable pump was used to vary the water velocity of water tunnel. In this testing, the plate samples only with steel mesh were selected as contrast sample for drag reduction calculating. The drag force of mesh films denoted as D_0_, the drag force of different films (ZnO film, ZnO/PDMS film, and ZnO/PDMS/n-octadecane film) denoted as D_p_, thus, the drag reduction coefficient of samples was calculated by the followed formula (1)


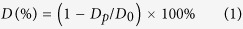


## Results and Discussion

**Figure 2:**
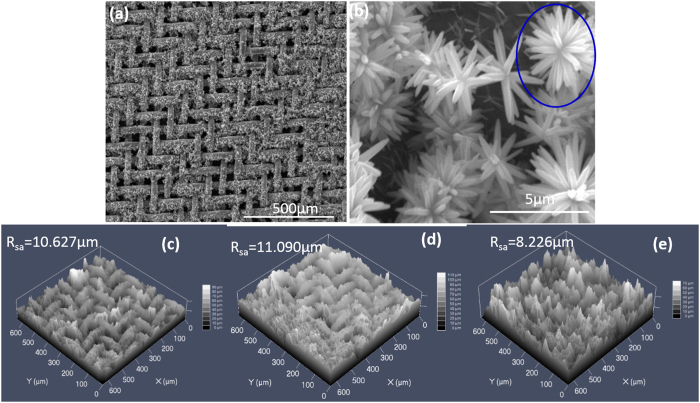
SEM images of the mesh covered with ZnO film (**a**,**b**) and 3-D morphology of mesh covered with ZnO film (**c**), ZnO/PDMS film (**d**) and ZnO/PDMS/n-octadecane film (**e**).

We designs a self-texturing ZnO/PDMS/n-octadecane film for drag reduction testing. The microstructure of ZnO film is observed by SEM, and the results are shown in Fig. 2. SEM image shows that the mesh all covered with dense ZnO film, High magnification image indicates that the ZnO is an acicular chrysanthemum and the ZnO clusters like a flowers. After modified by ZnO film, the mesh is better to storage liquid, such as high viscosity oil (n-octadecane), which can be attributed to the bionic structure. In addition, the high viscosity oil is similar to mucus on fish, thus, ZnO/PDMS/n-octadecane film can be used to simulate the compliant dolphin skin and mucus^32^,^33^.

3-D morphology of mesh covered with different films is characterized, as shown in Fig. 2. Large numbers peaks is existed on ZnO films, ZnO/PDMS and ZnO/PDMS/n-octadecane film. The roughness value of different film was about R_sa_10, and the ZnO/PDMS/n-octadecane film is smoother, which is because n-octadecane fills the low-lying area in ZnO/PDMS film.

**Figure 3:**
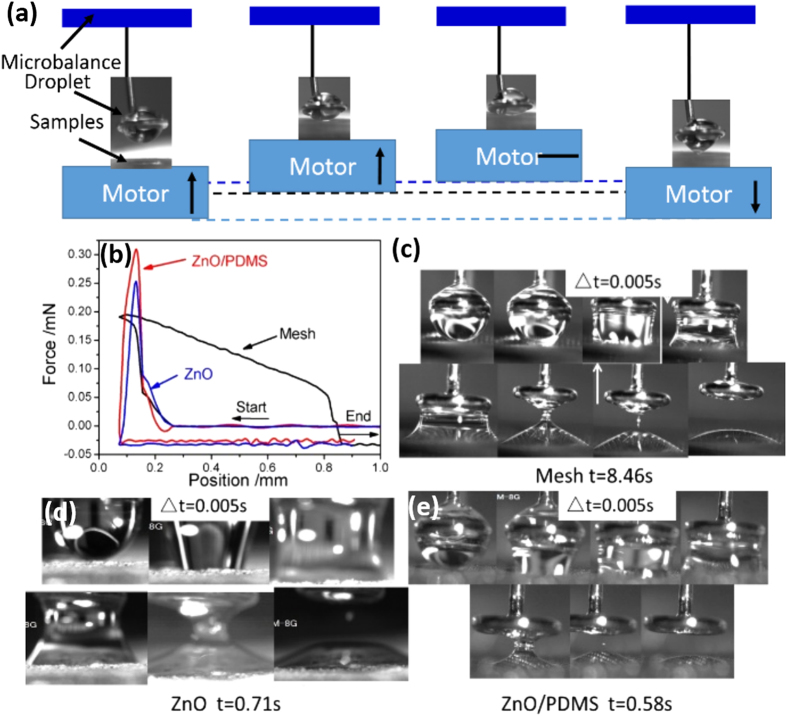
Schematic of the adhesive force testing process (**a**), adhesive force as a function of motor position of n-octadecane infltrated with diﬀerent film (**b**), Mesh (**c**), ZnO film (**d**), and ZnO/PDMS film (**e**).

The dynamic CA-measuring device is used to investigate the change of adhesive force of superoleophilic sample with n-octadecane, and a typical adhesive force curve of n-octadecane with different films is shown in Fig. 3. Prior to contacting with the suspended n-octadecane droplet, the change of weight was zero during the film samples moving toward n-octadecane droplet. Upon contacting the n-octadecane droplet, the force increases sharply because n-octadecane droplet is adsorbed by superoleophilic films. During contacting process, n-octadecane droplet is rapidly adsorbed, because the superoleophilic film sample is easily wetted by oil, such as n-octadecane.

The adhesive behaviors of n-octadecane with above three films indicates that the ZnO/PDMS film is easier wetted by n-octadecane. Furthermore, the time of wetting process shows that ZnO/PDMS is 0.58 s, but it is 8.46 s for mesh and 0.71 s for ZnO film. The result confirms that ZnO/PDMS film is more superoleophilic, which is better used to storage n-octadecane.

**Figure 4:**
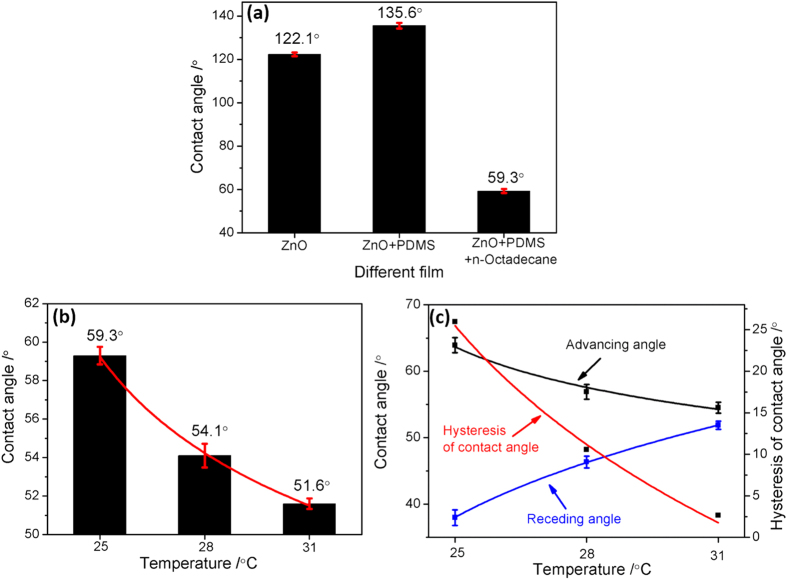
Contact angle evolution of different film (**a**), contact angle evolution (**b**), and hysteresis of contact angle evolution (**c**) of ZnO/PDMS/n-octadecane film at different temperature.

The wettability of ZnO, ZnO/PDMS, and ZnO/PDMS/n-octadecane film with water in air are shown in Fig. 4, respectively. In the liquid/air/solid three-phase system, water droplet with the volume of 3 μL is used to contact with film samples. The surface contact angle of different film indicates that ZnO and ZnO/PDMS film is hydrophobicity samples, and ZnO/PDMS/n-octadecane film is hydrophily samples. The change of contact angle indicates that the microstructure and surface energy of two type films are all remarkably different, thus, the drag reduction mechanism of three samples is also different.

The states of hydrophilic n-octadecane films is changed with temperature, the contact angle at solid, semisolid or liquid state is also varied with temperature. The change of contact angle, advancing angle, receding angle and hysteresis of contact angle of ZnO/PDMS/n-octadecane film at solid, semisolid, and liquid state is shown in Fig. 4(b,c). We knows that the melting temperature range of n-octadecane is 28.18 °C, thus, 25 °C, 28 °C, 31 °C is used to achieve the different state of n-octadecane. The results show that the contact angle of ZnO/PDMS/n-octadecane film decreases with temperature, indicating that the solid, semisolid, and liquid state of n-octadecane is corresponded to different contact angles. Furthermore, the advancing angle, receding angle and hysteresis of contact angle of ZnO/PDMS/n-octadecane film at different state shows that the advancing angle decreases and receding angle increases with temperature, thus, the hysteresis of contact angle decreases rapidly with temperature. The change of the hysteresis of contact angle indicates that the semisolid or liquid state is advantageous for the slipping of water droplet.

**Figure 5:**
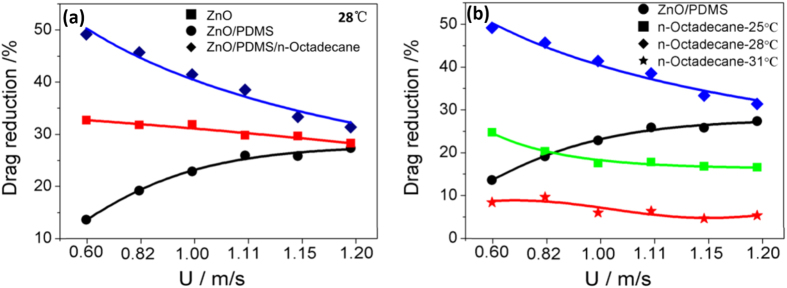
Drag reduction as a function of flow velocity for different film at 28 °C (**a**), ZnO/PDMS/n-octadecane film at different temperature (**b**).

Figure 5 shows the drag reduction against flow velocity for all different samples. Figure 5(a) indicates that all the film show a drag reduction when comparing with steel mesh at 0.6–1.2 m/s. The drag reduction coefficient of ZnO film is lower than ZnO/PDMS film and stable with flow velocity. The highest drag reduction is 49% for ZnO/PDMS/n-octadecane film at 0.6 m/s, and it decrease to 34% at 1.2 m/s, indicating that ZnO/PDMS/n-octadecane is more suitable for drag reduction, and the drag coefficient decreases with flow velocity.

The drag reduction coefficient against flow velocity of ZnO/PDMS/n-octadecane film at different state is shown in Fig. 5(b). It can be seen that ZnO/PDMS/n-octadecane film shows drag reduction at solid, semisolid, and liquid state, and the drag reduction coefficient of ZnO/PDMS/n-octadecane increases first and then decreases with temperature, the drag reduction coefficient of ZnO/PDMS/n-octadecane film is about 20% for solid state (25 °C), 40% for semisolid state (28 °C), and 8% for liquid state (31 °C). The results indicate that n-octadecane in semisolid state is effectively in drag reduction. In addition, the drag reduction of semisolid n-octadecane film is much better than of superhydrophobic ZnO/PDMS film at same flow velocity and temperature. Thus, the difference in drag reduction mechanism is the main reasons for drag reduction of superhydrophobic ZnO/PDMS and hydrophilic n-octadecane film.

**Figure 6:**
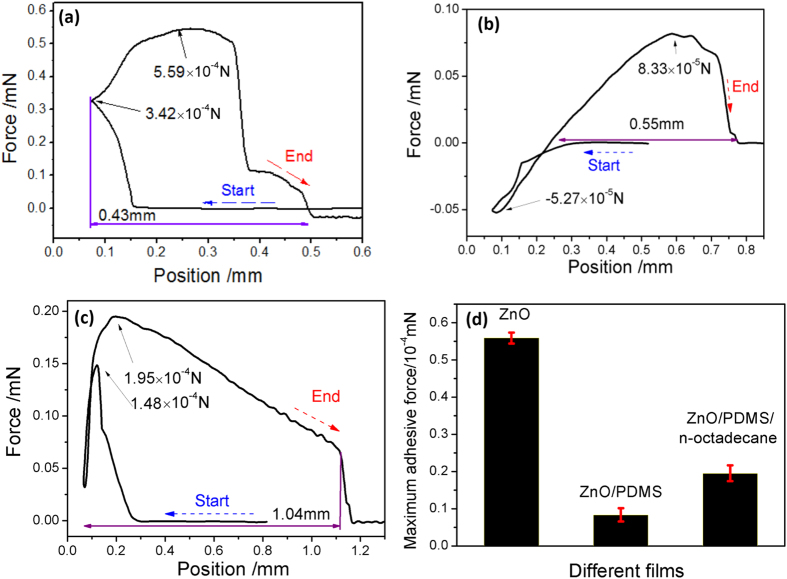
Adhesive force as a function of motor position for the ZnO film (**a**), ZnO/PDMS film (**b**), ZnO/PDMS/n-octadecane film (**c**) at 28 °C, and Maximum adhesive force of different films (**d**).

The adhesive behaviors of water with the above three films are shown in Fig. 6. For ZnO film (Fig. 6a), the maximum force between the samples and water is 5.59 × 10^−5^ N, which is lower than that of ZnO/PDMS films (8.33 × 10^−5^ N). The increased of adhesive force suggests that the adhesion role between water and supports sample is relatively large, and this results are according with the drag reduction of two film. Thus, the variation of adhesive behaviors supports the hypothesis that the drag reducing effects of superhydrophobic film is correlated with adhesive force, high adhesive force means that the water droplet is hard to move on the superhydrophobic film. In addition, the adhesive force of ZnO film is negative after detachment, indicating that part of water droplet remains on the substrate. Thus, the status of that whether water captured by the samples indicates that the film is good or bad for drag reduction. Furthermore, the drag force is not only affected by adhesion, such as surface texture, mucous layer or water flow all have significant effect on drag reduction. Thus, the maximum adhesive force of n-octadecane films at semisolid state is although higher than superhydrophobic ZnO/PDMS film, but the drag reduction coefficient of hydrophobic ZnO or ZnO/PDMS film are 28.2% and 26.5%, and it is 34.0% for hydrophilic n-octadecane film. The results show that the drag reduction mechanism of superhydrophobic film or hydrophilic n-octadecane are obviously different.

**Figure 7:**
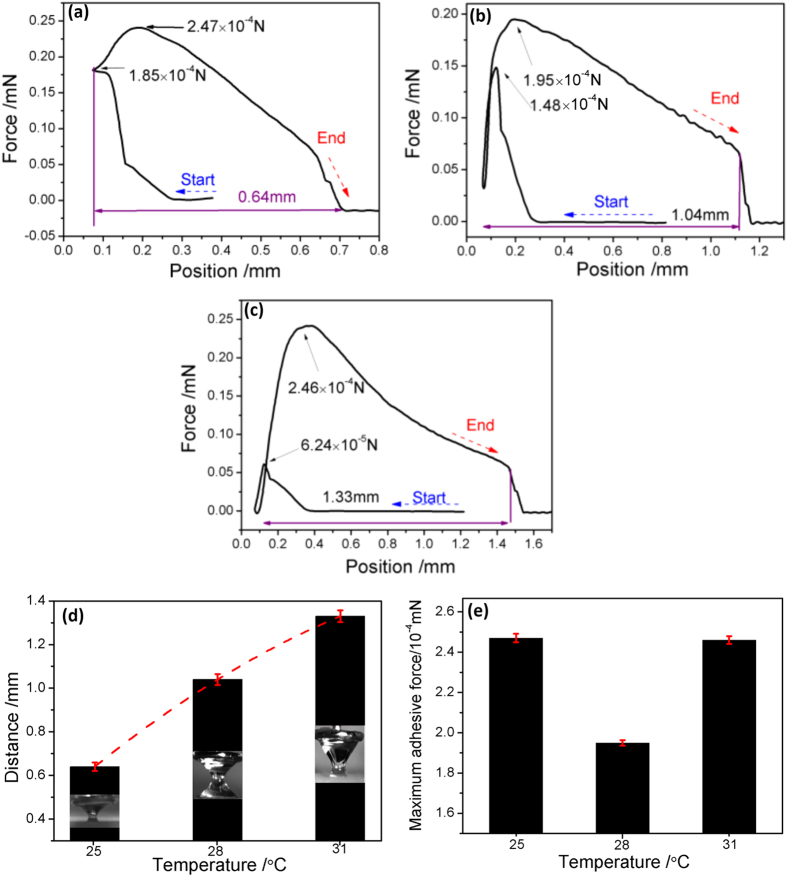
Adhesive force as a function of motor position for ZnO/PDMS/n-octadecane film at 25 °C (**a**), 28 °C (**b**), 31 °C (**c**), Distance (**d**), and maximum adhesive force (**e**).

The adhesive behaviors of water with ZnO/PDMS/n-octadecane film at different state are shown in Fig. 7. For n-octadecane film at solid state (25 °C), the maximum force is high to 2.47 × 10^−4^ N, indicating the adhesive force between the solid n-octadecane film and water is much high than superhydrophobic samples. In addition, the maximum force of semisolid and liquid state is 1.95 × 10^−4^ N and 2.46 × 10^−4^ N, displaying that the adhesive force between semisolid n-octadecane film and water is much lower than liquid state. Thus, low adhesive force may be one reasons for the lower drag of semisolid n-octadecane film.

**Figure 8:**
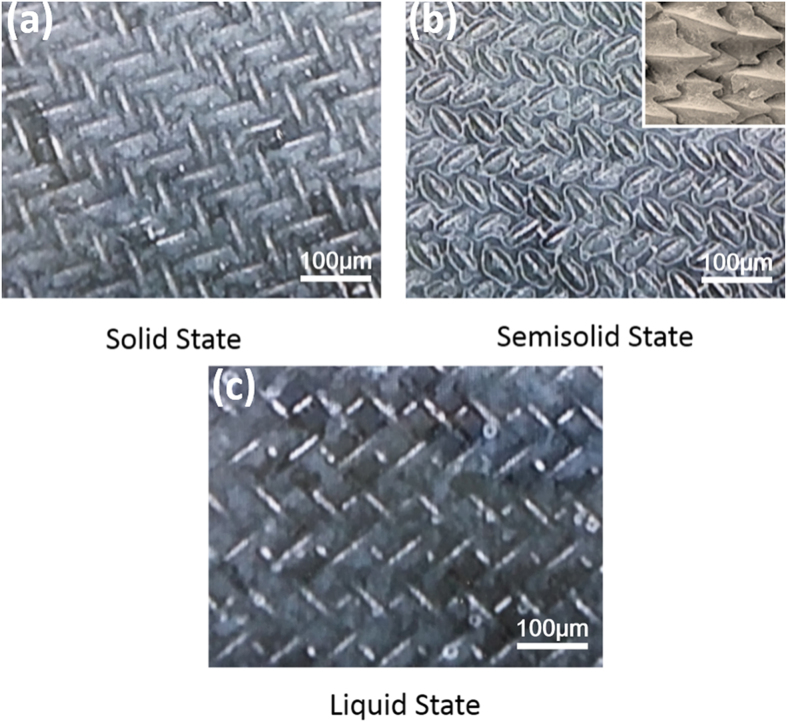
Microstructure of n-octadecane film (**a**) at 25 °C (**b**) 28 °C and (**c**) 31 °C.

Drag reduction of fish is closely connected with the microstructure of skin or the mucus layer on skin, thus, only adhesion but also microstructure or high viscosity mucus layer all have obviously impact on the drag reduction of ZnO/PDMS/n-octadecane films. In order to study the microstructure of n-octadecane film at different temperature, OM images of n-octadecane at 25 °C, 28 °C, 31 are shown in Fig. 8. At 25 °C, n-octadecane is all in solid state and with no liquidity, thus, the microstructure of n-octadecane film is similar to the morphology of mesh. At 28 °C it can be seen that the semisolid n-octadecane is consisted of solid and liquid phases. The melting part of n-octadecane is mainly located at the connection area of mesh and the solid part is in the gap between the wire mesh. OM image shows that the mesh of semisolid n-octadecane is similar to shark skin or dolphin skin and high viscosity liquid n-octadecane acts as fish mucus. The distribution of high viscosity liquid n-octadecane is self-textured along the water flow. Thus, the drag reduction of ZnO/PDMS/n-octadecane film at semisolid state can be attributed to the self-texturing compliant bionic structure and mucus layer. At 31 °C, melted n-octadecane become a liquid, the liquid n-octadecane shows low viscosity and it is easier flowed under water. Thus, semisolid n-octadecane is better to simulate the fish skin and mucus, and the ZnO/PDMS provides well effect on the store of high viscosity n-octadecane.

**Figure 9:**
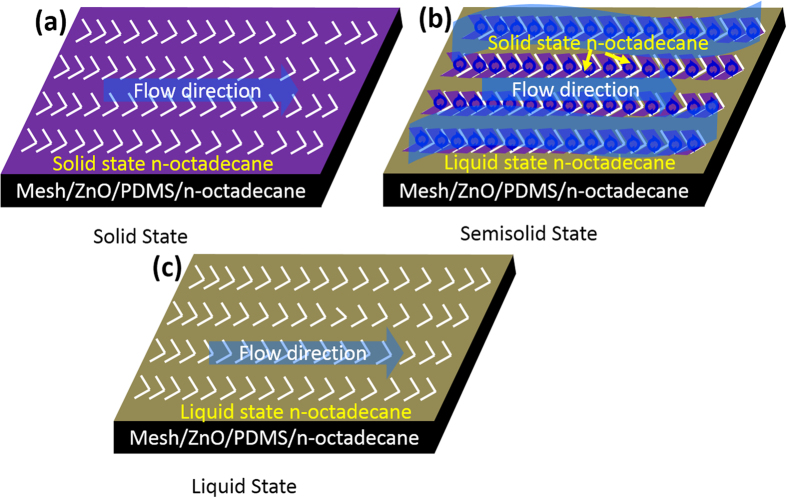
Illustration for the drag reduction mechanism of ZnO/PDMS/n-octadecane film at semisolid states.

The drag reduction mechanism of n-octadecane film is significantly different from hydrophobic samples, as shown in Fig. 9. As we known, the melting process of n-octadecane occurs in a certain temperature range, thus, three states of solid, semisolid and liquid state exists in the melting process of n-octadecane. For the semisolid states, n-octadecane films are composed of the liquid area and solid area. Hale provides conclusive evidence that n-octadecane is partly melted at semisolid, and the residual solid areas are disorderly distributed in liquid area^29^, as shown in Fig. 9. When water flows through the films, self-texturing surface likes a fish skin and mucus, thus it plays an obviously role in drag reduction. In addition, Sam indicates that the regular bulge will be formed on the skin surface during the dolphin swimming, and many micro-vortex appeared on the dolphin skin with regular bulge. Thus, the sliding friction of water flow can be replaced by rolling friction, and the drag of fish swimming decreases significantly^2^,^25^,^26^. According to this theory, it can be deduced that the semisolid n-octadecane is similar to the fish skin with regular bulge and mucus. Thus, the drag reduction action of n-octadecane at semisolid state may be consistent with the fish, which is benefited for ship, torpedo or other moving equipment in water.

Above all, the studies of n-octadecane film indicate a new way to reduce the drag actively. Not only the n-octadecane, such as the materials that surface morphology changed with PH, temperature, or sunshine can also be used to change the drag (increasing or decreasing). Thus, the design and application of self-texturing compliant bionic materials will play an important role in the field of drag reduction and the smart material will be widely used in this field.

## Conclusion

In summary, we have utilized the ZnO and PDMS mesh grafted with temperature-responsive n-octadecane to study the drag reduction of compliant bionic material. The states of n-octadecane changed with temperature, thus, the surface contact angle, advancing angle, receding angle, hysteresis of contact angle and adhesive force all varied obviously at solid state, semisolid state, and liquid state. For example, the surface contact angle and hysteresis of contact angle decreased with temperature, the adhesive force shown a lower value at semisolid state. In addition, the drag testing results shown that the hydrophilic n-octadecane film was more effectively in drag reduction than superhydrophobic ZnO/PDMS film, indicating that the drag reduction mechanism of n-octadecane film was significantly different with superhydrophobic film. Further research deduced that the semisolid state n-octadecane was like compliant fish skin with regular bulge and mucus. The sliding friction of water flow can be replaced by rolling friction, which is the main reason for drag reduction.

## Additional Information

**How to cite this article**: Liu, E. *et al*. Drag reduction through self-texturing compliant bionic materials. *Sci. Rep.* **7**, 40038; doi: 10.1038/srep40038 (2017).

**Publisher's note:** Springer Nature remains neutral with regard to jurisdictional claims in published maps and institutional affiliations.

## References

[b1] Cohen R. C., Cleary P. W. & Mason B. R. Simulations of dolphin kick swimming using smoothed particle hydrodynamics. Human movement science 31, 604–619 (2012). 21840077 10.1016/j.humov.2011.06.008

[b2] Pavlov V. Dolphin skin as a natural anisotropic compliant wall. Bioinspiration & biomimetics 1, 31 (2006). 17671304 10.1088/1748-3182/1/2/001

[b3] Bixler G. D. & Bhushan B. Bioinspired micro/nanostructured surfaces for oil drag reduction in closed channel flow. Soft Matter 9, 1620–1635 (2013).

[b4] Pastoor M., Henning L., Noack B. R., King R. & Tadmor G. Feedback shear layer control for bluff body drag reduction. Journal of fluid mechanics 608, 161–196 (2008).

[b5] Gyr A. & Bewersdorff H.-W. Drag reduction of turbulent flows by additives. Vol. 32 (Springer Science & Business Media, 2013).

[b6] Bhushan B. & Jung Y. C. Natural and biomimetic artificial surfaces for superhydrophobicity, self-cleaning, low adhesion, and drag reduction. Progress in Materials Science 56, 1–108 (2011).

[b7] Srinivasan S. . Drag reduction for viscous laminar flow on spray-coated non-wetting surfaces. Soft Matter 9, 5691–5702 (2013).

[b8] Ellis R. The dolphins’ secret. National Wildlife 22, 34–35 (1984).

[b9] Bixler G. D. & Bhushan B. Fluid Drag Reduction with Shark‐Skin Riblet Inspired Microstructured Surfaces. Advanced Functional Materials 23, 4507–4528 (2013).

[b10] Zhang D.-Y., Luo Y.-H., Xiang L. & Chen H.-W. Numerical simulation and experimental study of drag-reducing surface of a real shark skin. Journal of Hydrodynamics, Ser. B 23, 204–211 (2011).

[b11] Dou Z., Wang J. & Chen D. Bionic research on fish scales for drag reduction. Journal of Bionic Engineering 9, 457–464 (2012).

[b12] Liu K. & Jiang L. Bio-inspired design of multiscale structures for function integration. Nano Today 6, 155–175 (2011).

[b13] Fish F. E. & Hui C. A. Dolphin swimming–a review. Mammal Review 21, 181–195 (1991).

[b14] Alben S., Shelley M. & Zhang J. Drag reduction through self-similar bending of a flexible body. Nature 420, 479–481 (2002). 12466836 10.1038/nature01232

[b15] Gyorgyfalvy D. Possibilities of drag reduction by the use of flexible skin. Journal of Aircraft 4, 186–192 (1967).

[b16] Gad-el-Hak M. Compliant coatings for drag reduction. Progress in Aerospace Sciences 38, 77–99 (2002).

[b17] Gad-El-Hak M. In Flow Past Highly Compliant Boundaries and in Collapsible Tubes 191–229 (Springer, 2003).

[b18] Kramer M. O. Boundary layer stabilization by distributed damping. Journal of the Aerospace Sciences 27, 69–69 (1960).

[b19] Ellis R. The dolphin’ secret. National Wildlife 22, 34–35 (1984).

[b20] Carpenter P. W. Optimization of multiple-panel compliant walls for delay of laminar-turbulent transition. Aiaa Journal 31, 1187–1188, doi: 10.2514/3.11750 (1993).

[b21] Kramer M. O. Vol. 24 459–460 (AMER INST AERONAUT ASTRONAUT 1801 ALEXANDER BELL DRIVE, STE 500, RESTON, VA 22091, 1957).

[b22] Choi K. S. . In Proceedings of the Royal Society of London A: Mathematical, Physical and Engineering Sciences 2229–2240 (The Royal Society).

[b23] Blick E. F. & Walters R. R. Turbulent boundary-layer characteristics of compliant surfaces. Journal of Aircraft 5, 11–16 (1968).

[b24] Blick E. & Looney W. Skin-friction coefficients of compliant surfaces in turbulent flow. Journal of Spacecraft and Rockets 3, 1562–1564 (1966).

[b25] Ridgway S. H. & Carder D. Features of dolphin skin with potential hydrodynamic importance. Engineering in Medicine and Biology Magazine, IEEE 12, 83–88 (1993).

[b26] Ridgway S. H. & Carder D. A. In Sensory Abilities of Cetaceans 163–179 (Springer, 1990).

[b27] Caudwell D., Trusler J., Vesovic V. & Wakeham W. The viscosity and density of n-dodecane and n-octadecane at pressures up to 200 MPa and temperatures up to 473 K. International Journal of Thermophysics 25, 1339–1352 (2004).

[b28] Zhang X., Fan Y., Tao X. & Yick K. Fabrication and properties of microcapsules and nanocapsules containing n-octadecane. Materials Chemistry and Physics 88, 300–307 (2004).

[b29] Hale N. & Viskanta R. Photographic observation of the solid-liquid interface motion during melting of a solid heated from an isothermal vertical wall. Letters in heat and Mass Transfer 5, 329–337 (1978).

[b30] Hale N. & Viskanta R. Solid-liquid phase-change heat transfer and interface motion in materials cooled or heated from above or below. International Journal of Heat and Mass Transfer 23, 283–292 (1980).

[b31] Qiu X., Li W., Song G., Chu X. & Tang G. Fabrication and characterization of microencapsulated n-octadecane with different crosslinked methylmethacrylate-based polymer shells. Solar Energy Materials and Solar Cells 98, 283–293 (2012).

[b32] King M., Zahm J., Pierrot D., Vaquez-Girod S. & Puchelle E. The role of mucus gel viscosity, spinnability, and adhesive properties in clearance by simulated cough. Biorheology 26, 737–745 (1988). 10.3233/bir-1989-26406 2611367

[b33] Bernadsky G., Sar N. & Rosenberg E. Drag reduction of fish skin mucus: relationship to mode of swimming and size. Journal of fish biology 42, 797–800 (1993).

